# The Role of Animal Cognition in Human-Wildlife Interactions

**DOI:** 10.3389/fpsyg.2020.589978

**Published:** 2020-11-04

**Authors:** Madeleine Goumas, Victoria E. Lee, Neeltje J. Boogert, Laura A. Kelley, Alex Thornton

**Affiliations:** ^1^ Centre for Ecology and Conservation, University of Exeter, Cornwall, United Kingdom; ^2^ Animal and Veterinary Sciences, Scotland’s Rural College (SRUC), Midlothian, United Kingdom

**Keywords:** animal cognition, human-wildlife interactions, gaze sensitivity, individual recognition, class-level recognition, categorization, generalization, behavioral flexibility

## Abstract

Humans have a profound effect on the planet’s ecosystems, and unprecedented rates of human population growth and urbanization have brought wild animals into increasing contact with people. For many species, appropriate responses toward humans are likely to be critical to survival and reproductive success. Although numerous studies have investigated the impacts of human activity on biodiversity and species distributions, relatively few have examined the effects of humans on the behavioral responses of animals during human-wildlife encounters, and the cognitive processes underpinning those responses. Furthermore, while humans often present a significant threat to animals, the presence or behavior of people may be also associated with benefits, such as food rewards. In scenarios where humans vary in their behavior, wild animals would be expected to benefit from the ability to discriminate between dangerous, neutral and rewarding people. Additionally, individual differences in cognitive and behavioral phenotypes and past experiences with humans may affect animals’ ability to exploit human-dominated environments and respond appropriately to human cues. In this review, we examine the cues that wild animals use to modulate their behavioral responses toward humans, such as human facial features and gaze direction. We discuss when wild animals are expected to attend to certain cues, how information is used, and the cognitive mechanisms involved. We consider how the cognitive abilities of wild animals are likely to be under selection by humans and therefore influence population and community composition. We conclude by highlighting the need for long-term studies on free-living, wild animals to fully understand the causes and ecological consequences of variation in responses to human cues. The effects of humans on wildlife behavior are likely to be substantial, and a detailed understanding of these effects is key to implementing effective conservation strategies and managing human-wildlife conflict.

## Introduction

**Figure 1:**
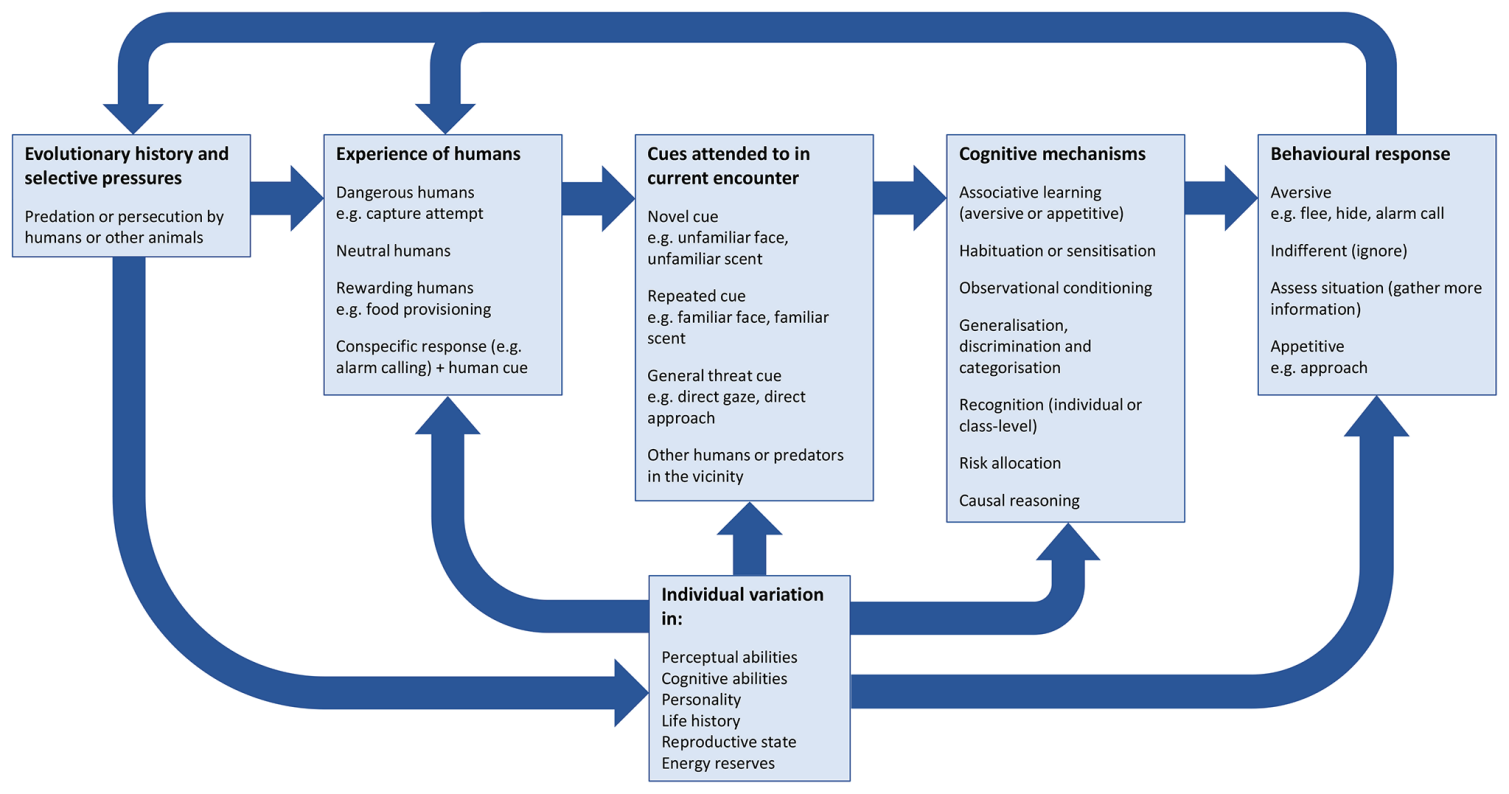
Factors that may affect wild animals’ behavioral responses during interactions with humans.

Humans have had a negative impact on other animals for millennia (Barnosky et al., 2004) and, with the human population continuing to grow (Roser et al., 2013), wild animals may encounter humans with increasing frequency. Few wild animal species are unaffected by humans, and human activity undoubtedly creates huge and varied selection pressures (Sih et al., 2011). Wild animals must avoid being hunted and persecuted, make foraging decisions in the presence of humans and select breeding sites in a human-dominated landscape. Additionally, habitat destruction can bring animals into close proximity to humans, where competition for food and space often leads to conflict (Pirta et al., 1997). As humans are a key driver of wildlife declines, understanding the behavioral and cognitive processes that shape wild animals’ responses to humans is likely to be important in mitigating the detrimental effects of human activity. To successfully navigate encounters with humans, animals rely on a wide range of cognitive processes, as they must perceive and attend to relevant cues, integrate this information with previous experience, and mount the appropriate behavioral response (Figure 1).

While interactions between humans and domesticated animals have been relatively well-studied (e.g., Hare et al., 2002; Miklósi and Soproni, 2006; Proops and McComb, 2010; Nawroth et al., 2015), comparatively little research has focused on how free-living, wild animals respond to and interact with humans. Furthermore, as domesticated animals have been selected for docility and sociability toward humans (Wilkins et al., 2014; VonHoldt et al., 2017), the responses of such animals, even when feral, are likely to differ substantially from those of species with no evolutionary history of domestication. For instance, many wild species have a history of being hunted or persecuted, and avoidance of humans may be crucial for their survival. However, others have no such history and humans present a novel threat. Perhaps the best known case of human-mediated extinction in modern history is that of the dodo (*Raphus cucullatus*), whose naïveté to predators rendered the species vulnerable to exploitation by humans (Cheke, 2004). To this day, wild animals risk following the same fate (Ripple et al., 2019). Unless animals have the behavioral flexibility to accommodate anthropogenic change, it is likely that they will be disadvantaged (Lowry et al., 2013).

In this review, we consider human-wildlife interactions from the perspective of wild animals by examining how they perceive and respond to humans. We focus predominantly on studies of free-living animals and those brought into captivity temporarily for the purpose of study. Although animals raised in captivity can provide interesting insights into the potential cognitive abilities of their free-living counterparts, it is likely that extensive experience of humans modifies their behavior. We consider the cognitive challenge of discriminating between humans that pose differing levels of threat and responding appropriately. The factors that may drive individual differences in wild animals’ responses to humans are also considered. The range of cues and the cognitive mechanisms that wild animals use to inform their responses to humans are not fully understood, and it is likely that humans affect animal behavior in ways that are not yet realized. We conclude by emphasizing the important role of animal cognition research in reducing human-wildlife conflict and improving conservation outcomes.

## How do Wild Animals Respond to Humans as a Species?

It is often unknown whether wild animals have evolved specific responses to humans, and whether flexible responses to humans are human-specific. Humans may be seen in a similar way to other animals in the area, which could be as a predator or an insignificant part of the environment. While fear of humans may have a heritable component (Carrete et al., 2016), animals are able to learn to fear certain stimuli (though not all; Cook and Mineka, 1990). Understanding how wild animals perceive humans may help minimize wildlife disturbance and allow the implementation of more effective conservation strategies.

Studies of wild animals on islands that have been free of human activity throughout their evolutionary history show that, at least for some species, it may be difficult to learn to recognize novel predators such as humans. These islands also tend to be free of other large, terrestrial predators and, when predators are introduced, the naïve island species often lack an appropriate antipredator response (Sih et al., 2010). The vast majority of mammalian and avian extinctions in recent history have been island species (Johnson and Stattersfield, 1990; Ceballos and Brown, 1995), which may be due at least in part to their naïveté. Island species tolerate a closer approach by humans in comparison to closely related species in mainland areas, and the remoteness of islands further increases tolerance (Cooper et al., 2014). This is likely due to a historical lack of mammalian predators. For example, the Galápagos Islands have never been in contact with the mainland and large mammals have arrived only recently. Even after experiencing sustained chasing by a human, marine iguanas (*Amblyrhynchus cristatus*) did not show an increase of the stress hormone cortisol (Rödl et al., 2007). Additionally, while iguanas’ heart rate increased upon seeing a native avian predator, they did not initiate a cardiovascular stress response at the sight of an approaching human, despite previously having experienced experimental capture (Vitousek et al., 2010).

Species that have evolved alongside large terrestrial predators indicate that wild animals may exhibit a generalized antipredator response and that current predation pressure may increase sensitivity to humans. For example, the presence of mammalian predators increases fear of humans in tammar wallabies (*Macropus eugenii*, Blumstein, 2002), and double-banded plovers (*Charadrius bicinctus*) flush sooner from humans in areas where domestic cats are present (St Clair et al., 2010). Predation pressure combined with non-predatory disturbance by humans may be sufficient to maintain antipredator responses to humans (Frid and Dill, 2002; Figure 1).

Wild animals that frequently encounter humans are likely to adjust their behavior in response to human disturbance. Animals may avoid areas where human disturbance is high (Dyer et al., 2001), but, if humans are not dangerous, they may remain in the area and habituate to human presence (Walker et al., 2006). In some cases, habituation to humans may produce negative consequences: for instance, there are concerns that great ape populations that are habituated to the presence of researchers may be more likely to enter croplands and come into conflict with local people (Hockings et al., 2015). Moreover, a decrease in escape response in areas where prolonged human disturbance occurs does not necessarily mean that animals perceive humans to be a lesser threat: animals may have little choice but to forage during times of high human disturbance and may adjust their responses according to temporal variation in human density, known as “risk allocation” (Lima and Bednekoff, 1999; Ferrari et al., 2009; Rodriguez-Prieto et al., 2009). Fear responses to humans are also affected by spatial variation in risk. For example, elk (*Cervus elaphus canadensis*) increase their vigilance in areas where hunting by humans occurs; this leads to a decrease in time spent feeding (Ciuti et al., 2012b). Some male red deer (*C. elaphus*) avoid hunting areas during the hunting season, despite these areas containing preferred forage, and ultimately have a better survival rate (Lone et al., 2015). Furthermore, a later study found that the feces of red and roe deer (*Capreolus capreolus*) contained higher stress hormone concentrations in areas where the main predators are humans rather than large carnivores (Zbyryt et al., 2018), indicating that these species perceive humans to be different from, and potentially more dangerous than, other predators. These studies highlight the trade-off many wild animals face between feeding and avoiding predation by humans, and the importance of making correct decisions about when and where to forage.

Human disturbance may have many long-term effects. If the presence of humans is stressful, this may be particularly problematic for species of conservation concern. Although some species habituate to human presence, others appear to become sensitized to it, with higher human disturbance causing an increase in stress responses. Even within groups of closely related species and when the nature of disturbance is similar, contrasting effects of human activity are evident. For example, while Magellanic penguins (*Spheniscus magellanicus*) exposed to tourism had lower stress hormone concentrations than their undisturbed counterparts (Walker et al., 2006), the opposite was true for yellow-eyed penguins (*Megadyptes antipodes*, Ellenberg et al., 2007). The stress induced by tourism resulted in lower reproductive success for the yellow-eyed penguins, a species already listed as endangered (Ellenberg et al., 2007). The apparent failure of some species to habituate to human presence is likely to be a key issue for conservation.

Responding to novel humans based on previous encounters with other humans requires some level of generalization of what was learned during these prior experiences: animals can only habituate to humans if they identify humans as being members of the same category, despite each human appearing different. Likewise, associations made during encounters with dangerous or rewarding humans are likely to influence later responses to humans (Figure 1). The degree to which wild animals generalize from their previous encounters, and their ability to discriminate between classes and individuals, depends on the cues that are attended to (see “Categorization of humans and class-level recognition” and “Individual recognition of humans”).

### Do Wild Animals Perceive Humans as Causal Agents?

The extent to which wild animals respond flexibly to humans may be influenced by their causal understanding of how humans interact with the environment. While responding to observable cues may often be sufficient, inferring that humans are capable of causing certain events may aid in modulating appropriate behavior. If wild animals are able to understand relationships between cause and effect, it may increase their ability to attend to relevant cues and ignore those that have no consequences. Evidence for an ability to recognize humans as causal agents has so far been controversial. Taylor et al. (2012) tested whether New Caledonian crows (*Corvus moneduloides*) differentiated between a stick that was moving because a human had entered a hidden location (a “hidden causal agent,” or HCA) from which the stick emerged, and a stick that appeared to be moving without human intervention (an “unknown causal agent,” or UCA). The stick was placed next to a feeder, such that crows could be hit if they fed while the stick was moving. The crows inspected the hole where the stick had come from less often, and were more inclined to feed, after the human had left the hide, while they were far more cautious after the UCA trials. The authors concluded that these results show that the crows were able to infer that the human caused the stick to move, and thus reasoned that it was safe to forage once the human had left the hide, while stick movement in the UCA trials was unpredictable. However, the authors presented all the crows with the HCA condition first, which means that the results might be confounded by increased test subject experience. This and other issues (see Boogert et al., 2013 and Dymond et al., 2013), mean that this result should be interpreted with caution.

In an experiment where North Island robins were given the choice of pilfering food in front of one of two humans, robins were more likely to avoid a human whose limbs were visible, a response that the authors suggest indicates reasoning about “capability” (Garland and Low, 2016). However, this trend was only observed when presented in combination with other modifications, such as stimulus size/shape and experimenter facial covering. Furthermore, if an understanding of capability is to be tested, the “incapable” human should be truly incapable of approaching the subject, and in such a way that is clearly observable. Despite these confounds, the general experimental setup seems suitable for tests of causal reasoning in habituated wild animals. A laboratory study with a similar design and sufficient controls indicated that captive chimpanzees (*Pan troglodytes*) were not able to reason about human limb capability: chimpanzees begged for food from humans who were physically unable to use their limbs to provide food, and did not learn over successive trials (Vonk and Subiaul, 2009). Although research on causal reasoning in non-human animals continues, there is currently little evidence that non-human animals have a robust understanding of the relationships between cause and effect (see Penn et al., 2008; Schloegl and Fischer, 2017 for reviews). It would be interesting to establish whether an inability to perceive humans as causal agents affects species’ susceptibility to the negative effects of human activity.

## The Nature of Human-Wildlife Interactions

Humans are an unusual species in that they can take a wide range of roles in their interactions with heterospecifics. Humans can present a unique challenge to wild animals, as different humans can pose different levels of threat: while many people ignore wild animals, some people kill them, and others actively feed them. Wild animals that live alongside humans would benefit from being able to discriminate between humans taking these vastly different roles (Figure 1). Here, we describe how the different roles that humans take affect animal behavior, before considering the cognitive mechanisms that potentially allow wild animals to overcome the challenge of distinguishing between them.

### Dangerous Humans

Humans pose a threat to wild animals for a range of reasons. Humans may act as predators, killing animals for food (Ripple et al., 2015), sport (Loveridge et al., 2007), or even for conservation purposes (Russell et al., 2016). They may also act as competitors and kill animals to prevent or reduce consumption or damage of resources. Large carnivores such as lions (*Panthera leo*) are often killed to prevent predation of domesticated animals (Woodroffe and Frank, 2005), while herbivorous mammals and birds are commonly targeted for consuming crops (Gebhardt et al., 2011; Ango et al., 2017). Animals targeted by lethal practices may benefit from showing heightened fear of humans. For example, an experimental study found that black-billed magpies (*Pica hudsonia*) flew away sooner from an approaching human in rural agricultural areas, where they are persecuted, than in rural parks, where they face no such persecution (Kenney and Knight, 1992). A long-term study found that coyotes (*Canis latrans*) became more active during the daytime after intense persecution from humans had ended (Kitchen et al., 2000), and a recent meta-analysis indicated that mammals in areas of high human disturbance have become more nocturnal compared with conspecifics in areas where human disturbance is lower (Gaynor et al., 2018). The type of persecution animals face also appears to be important: crows (*Corvus macrorhynchos* and *Corvus corone*) are more wary of humans in areas where they are shot rather than cage-trapped, perhaps because associations between humans and dead conspecifics are formed more easily in the former case (Fujioka, 2020). A particularly striking example of how wild animals might learn to evade human predation comes from Diana monkeys (*Cercopithecus diana*), which usually respond to predators by alarm calling and approaching. Human hunters have taken advantage of this by imitating calls of predators and distressed prey. Monkeys in areas where poaching occurs have an increased ability to distinguish between imitations by humans and real alarm calls, and subsequently call less, compared to monkeys in areas where there is no poaching (Bshary, 2001). These studies indicate that individuals of targeted species are able to respond flexibly to direct threats posed by humans. Moreover, these examples show how human perceptions and differences in cultural practices can ultimately shape wild animal behavior.

### Neutral Humans

Many humans present no direct threat to wild animals. A neutral human will either ignore wild animals or observe them from afar, and will not interfere with their behavior. An example of a neutral human could be someone who allows wild animals to live close by without either deterring or encouraging them. If an animal only ever encounters neutral humans, they are likely to exhibit behavior that differs from that of animals with experience of dangerous humans. Responding aversively to humans that do not present a threat is suboptimal as it is likely to entail unnecessary energetic costs and reduced feeding time (Ydenberg and Dill, 1986). Animal populations that experience high human disturbance, such as those in urban areas, are often more tolerant of humans than are those in areas of lower human disturbance (Samia et al., 2015). This could be a result of habituation to repeated non-threatening encounters, or reflect population-level differences in tolerance that enable certain individuals to settle in areas where they will be frequently disturbed (Blumstein, 2016).

Of course, humans can intend to be neutral but their behavior could have unintentional consequences that create positive or negative outcomes for wild animals, e.g., through accidentally dropping food or littering. Additionally, whether or not wild animals make aversive or appetitive associations with humans in general can be out of an individual human’s control. As animals are able to associate events with neutral environmental stimuli (Cassens et al., 1980), wild animals may perceive humans as “dangerous” or “rewarding” irrespective of whether that human caused a particular outcome. How animals view neutral humans may also be affected by their previous experiences with other people, and the extent to which they generalize or discriminate between individual humans.

### Rewarding Humans

Although many interactions with humans appear to be neutral or negative from the perspective of wild animals, interacting with humans can also be advantageous. Many humans purposefully provide care to wild animals, including through direct feeding interactions (Marion et al., 2008). While such close contact can carry a risk of harm to both humans and wild animals (e.g., from disease and aggression; Orams, 2002), such interactions provide at least short-term benefits and often result in attraction to humans (Sabbatini et al., 2006; Donaldson et al., 2010). Humans also provide food indirectly, for example by accidentally dropping food during picnics, and may thus be associated with reward (Marion et al., 2008). Relatively little research has focused on the effects of “rewarding” humans on wild animal behavior. However, risk-sensitive foraging theory predicts that the cost of failing to respond appropriately to humans in dangerous roles (i.e., by fleeing or hiding) would outweigh the benefits of being attracted to humans in a rewarding role: even if the risk of being killed is low, the risk of starving from a lack of extra food is likely to be far lower (McNamara and Houston, 1992).

It is even possible for humans to have a mutualistic relationship with wild animals, where both parties gain measurably from the interaction. In parts of Africa, for example, humans forage for honey alongside greater honeyguides (*Indicator indicator*), which feed on bees’ wax and larvae. These brood-parasitic birds are unable to access bees’ nests and actively solicit human cooperation (Isack and Reyer, 1989). Honey hunters can also attract a honeyguide by making a specific call (Isack and Reyer, 1989). As honey hunters report that juvenile honeyguides, which are raised by other species, initiate foraging trips, it is likely that this relationship has evolved through selection (Spottiswoode et al., 2016). The observation that they do this before responding to the call indicates that there is also likely to be an important role for learning, particularly as honeyguides respond to the specific calls of the honey hunters in their local area (Spottiswoode et al., 2016). Whether honeyguides learn these calls through individual experience or socially from the responses of conspecifics is currently unknown. It is plausible that a honeyguide could learn to associate a honey-hunting call with the subsequent reward of food if honey hunters call while following the honeyguide to the bee’s nest. Examples such as this exemplify why some wild animals benefit from being attracted to human cues.

## How do Wild Animals Distinguish Between Dangerous and Neutral Humans?

Animals may respond differently to different groups of humans and exhibit a specific response only to humans displaying a particular cue, such as a distinctive item of clothing (e.g., Bates et al., 2007). If only a certain behavior or type of human represents a threat, animals will benefit from attending to these cues rather than those of neutral humans (Figure 1). Animals may respond to cues that are threatening regardless of the species displaying them if they are intrinsically associated with negative outcomes; these cues may or may not require learning. Examples of such general threat cues that affect wild animals’ behavior include direct gaze (discussed below), direct approach (Burger and Gochfeld, 1981), and a fast approach speed (Cooper et al., 2003). Wild animals may also learn to attend to cues that are specific to humans. Here we discuss cues that have been well-studied, but there are potentially many different types of cue that animals could use to inform their responses.

### Gaze Direction

Animals may use the direction of human gaze to identify and avoid dangerous humans. Gaze direction is an indication of where attention is directed, and human gaze direction is likely to be particularly discernible as humans have forward-facing eyes. Additionally, humans have visible white sclerae which, contrasted against the darker irises, potentially make the direction of their gaze more conspicuous than that of other mammals (Kobayashi and Kohshima, 1997). Gaze aversion, whereby animals exhibit a fearful response to another’s eye direction, appears to be taxonomically widespread among vertebrates and likely functions as a means of avoiding predation and altercations with competitors (see Davidson et al., 2014; Davidson and Clayton, 2016 for reviews of gaze sensitivity). Using gaze direction as a cue should enable animals to attend to dangerous or aggressive individuals in the environment while ignoring those that do not pose a threat. Indeed, wild animals of a wide range of species respond differently when a human is looking at them compared to looking away; they typically flee sooner (e.g., Burger et al., 1992; Eason et al., 2006; Bateman and Fleming, 2011; Clucas et al., 2013; Cooper and Sherbrooke, 2015), or take longer to approach food (Carter et al., 2008; Garland et al., 2014; Goumas et al., 2019) or their nests (Watve et al., 2002) when exposed to direct human gaze.

Animals may not necessarily respond aversively to human gaze in all contexts. Being approached by a human could be perceived as a predation attempt, whereas a human sitting passively while directing their gaze at an animal may have no such connotations. It may even be possible for wild animals to come to associate direct human gaze with reward. In cases of wildlife feeding, for example around duck ponds, human gaze may be appetitive rather than aversive, as a human is likely to direct food toward an individual it is looking at. However, to our knowledge, there has been no research on whether wild animals respond appetitively to human gaze. Interestingly, in a study of hand-raised, captive jackdaws, von Bayern and Emery (2009) found that test subjects only responded aversively to human gaze, measured by latency to retrieve food, when the human was unfamiliar to them. Whether free-living animals adjust their behavior in this manner has not been tested.

Gaze aversion experiments have not always distinguished between head direction and eye direction, but a response to head direction is not necessarily indicative of a reaction to eyes. In humans and other predators, head direction may be a good proxy for eye direction, and is potentially more salient, and therefore may be a useful cue for wild animals to use. However, using a cue that is only sometimes informative is not optimal. Hampton (1994) showed that captive house sparrows (*Passer domesticus*) attempted to escape most often when his head was facing them rather than turned away, regardless of eye direction. Some studies have found that several other passerine species do appear to pay attention to eyes specifically (American robins *Turdus migratorius*, Eason et al., 2006; European starlings *Sturnus vulgaris*, Carter et al., 2008; American crows *Corvus brachyrhynchos*, Clucas et al., 2013; North Island robins *Petroica longipes*, Garland et al., 2014).

Responses to eye direction invoke the question of whether wild animals have the ability to take another’s perspective. If animals are able to understand that other individuals have a different viewpoint, they may be able to better predict their behavior. Do animals that exhibit aversion to direct gaze understand that they are being watched? It could certainly explain why these individuals are fearful, but a “Theory of Mind” explanation is not necessary to account for the observed behavior, if, for example, eyes are inherently aversive or animals learn to associate direct gaze with a predation attempt. Studies where the experimenters direct their attention toward an object, rather than the test subject (e.g., Carter et al., 2008), suggest that wild animals of some species may not simply be reacting to the presence of eyes and are instead able to follow human gaze. This has been demonstrated in captive corvids and primates (e.g., common ravens *Corvus corax*, Bugnyar et al., 2004; gibbons *Hylobates* spp. Liebal and Kaminski, 2012).

While laboratory experiments indicate that corvids can take the perspective of conspecifics and may thus have a Theory of Mind (Dally et al., 2010; Bugnyar et al., 2016), very few studies have attempted to address the question of whether free-living wild animals understand the perspective of a human observer. Watve et al. (2002) devised an experiment that made use of visual barriers near the nests of green bee-eaters (*Merops orientalis*). The experimenter could take one of two positions when the focal bird was on a nearby perch, ready to enter the nest to feed its chicks. In one position, the experimenter could see the bird but not the nest; in the other, both could be seen. The bee-eaters made more visits to the nests and had a shorter approach latency when the experimenter’s view of the nest was obstructed, implying that the birds were not simply reacting to their view of the experimenter. However, the experimenter was looking at the bird rather than the nest. It is therefore unclear whether the bee-eaters were simply reacting to the experimenter watching them as they approached the nest, and were deterred by direct gaze. Stronger evidence for perspective-taking might be provided by a study where the experimenter measures the bird’s latency to leave the perch before entering the nest, while keeping their gaze directed at the nest.

The widespread nature, early-life presence and clear utility of gaze aversion have led to the assumption that such responses to gaze are “innate” (Coss, 1979; Shepherd, 2010). We interpret “innate” in this context to mean that animals do not require prior experience of gaze stimuli in order for gaze aversion to manifest. Although this may be a parsimonious explanation for its documented presence in several vertebrate classes, few studies have actually attempted to address this question. While several species show aversive responses to two horizontally-positioned eye-like stimuli early in development (ray-finned fishes: Coss, 1978; Altbäcker and Csányi, 1990; Miklósi et al., 1995; chickens *Gallus gallus*: Scaife, 1976; Jones, 1980), whether or not experience is required to mediate these responses is unclear and may be species-specific. For example, jewel fish (*Hemichromis bimaculatus*) that were deprived of seeing eyes or eye-like stimuli during early life showed an aversive response to two horizontal eye spots, whereas fish that were raised in the presence of conspecifics did not (Coss, 1979). Conversely, bobwhite quails (*Colinus virginianus*) raised without exposure to human faces tended to ignore the direction of human gaze, whereas those previously exposed to them avoided areas where a human was looking (Jaime et al., 2009). Without further studies that begin at birth or hatching, and control for exposure to all eyes or eye-like stimuli, it is impossible to conclude that gaze aversion is innate. There is some evidence that attention to eyes or eye-like stimuli may be innate by our definition (see e.g., Batki et al., 2000; Sewards and Sewards, 2002 for evidence from human neonates and other amniotes), and this may facilitate early development of gaze aversion. An evolved mechanism for attending to eye-like stimuli, and an ability to learn quickly, would provide animals with the capacity to use gaze cues without the need for perspective-taking.

### Categorization of Humans and Class-Level Recognition

The ability to categorize humans into groups based on shared features may allow animals to respond appropriately according to the risk associated with different groups. This is likely to be particularly important in areas where different groups of people pose different levels of threat. For example, the same area might be inhabited by some groups of people who commonly engage in hunting or kill wild animals to protect resources, while other people may not pose a threat. To categorize a human usefully, an animal must be able to discriminate between different classes of humans by attending to relevant cues, shared only by members of a single class, and ignoring uninformative cues. Distinguishing between dangerous and neutral classes of humans is likely to occur through associative learning, whereby animals associate the cue with an aversive action. Being able to recognize a member of a class (“class-level recognition”) requires that animals remember the cue and its association in later encounters.

Wild animals’ ability to categorize humans according to the level of threat they pose is beautifully illustrated by a series of experiments conducted in Amboseli National Park in Kenya. There, African elephants (*Loxodonta africana*) compete with domesticated animals for food and water and occasionally kill humans (Browne-Nuñez, 2011). This creates conflict with Maasai pastoralists, who spear elephants in retaliation (Browne-Nuñez, 2011). In contrast, the sympatric Kamba people pose relatively little threat to elephants (Bates et al., 2007). In an experiment to test whether elephants in Amboseli differentiate between the two groups of people, Bates et al. (2007) exposed free-living elephants to garments that had been worn by Maasai and Kamba men and assessed whether the scent of the garments affected their behavior. They also tested whether elephants could use visual cues to identify groups: Maasai people typically wear distinctive red clothing so the researchers measured elephants’ reactions to red vs. white unworn cloths. Elephants directed aggressive displays toward the red cloth at a higher frequency than they did towards the white cloth. They also moved faster and further away from Maasai-worn cloth than Kamba-worn cloth upon detecting the scent. Furthermore, the elephants responded similarly to the Maasai-worn cloth whether or not they had individual experience of being hunted by Maasai men, which indicates that elephants’ responses to threatening cues can be facilitated by social learning.

A subsequent experiment by McComb et al. (2014) tested elephants’ ability to differentiate between Maasai and Kamba people based on the sound of their voices. The researchers used playbacks of Maasai and Kamba men speaking the same words in their respective languages. The elephants were more likely to spend time sniffing and bunching up closely together (a defensive behavior) when they heard a Maasai man’s voice compared to a Kamba man’s voice. Additionally, elephants were more likely to retreat from the voices of Maasai men than those of Maasai women or boys: Maasai women and boys pose little threat to elephants. The elephants still responded with defensive behavior more frequently to the men’s voices than the women’s voices even after the pitch had been altered to resemble that of the opposite sex, suggesting that the acoustic cues they use to differentiate Maasai men from other groups are very subtle. Together, these experiments demonstrate that elephants can discriminate between threatening and non-threatening groups of people based on visual, olfactory and acoustic cues.

Visual cues may be particularly useful for wild animals being hunted, as hunters are likely to minimize the amount of sound they make. For example, a study of Poeppig’s woolly monkeys (*Lagothrix poeppigii*) in the Ecuadorian Amazon indicated that hunting pressure may affect this species’ responses to humans carrying objects and displaying behavior associated with danger (Papworth et al., 2013). Researchers simulated the appearance and behavior of individuals from groups of people that monkeys in the area were likely to have encountered previously: hunters, who regularly kill monkeys; gatherers, who collect resources on the ground and pose little threat to monkeys; and researchers, who usually passively observe monkeys. Observers recorded the change in behavior of the monkeys after detecting humans acting in each experimental condition and compared sites where monkeys were known to face low and high hunting pressure. In response to seeing a “hunter,” monkeys at both sites made fewer vocalizations, reduced their visibility and moved away, whereas their responses to the other conditions were mixed. While this experiment does not allow conclusions to be drawn about whether it is human behavior, objects, or the combination of these cues that are important in affecting woolly monkey behavior, it adds to the evidence that free-living animals may be able to distinguish dangerous from non-dangerous groups of people based on classifiable visual cues. Future research that assesses the relative importance of human behavior and associated objects would increase our understanding of the cues that wild animals use to infer the level of risk posed by different groups of people.

## Individual Recognition of Humans

While being able to classify humans into groups may be an effective way to evade danger, it will not always be possible to group humans usefully. Humans that may appear very similar can act very differently. In places where wild animals repeatedly encounter humans that exhibit consistent inter-individual differences in the level of threat they present, being able to accurately identify individual humans would facilitate avoiding risky encounters with dangerous individuals (Figure 1). Conversely, responding fearfully to humans that do not present a threat may lead to reduced feeding opportunities and increased movement, both of which would incur an energetic cost (Ydenberg and Dill, 1986); therefore, responding appropriately to those people who are known to be threatening or rewarding could be advantageous.

In order to recognize an individual, an animal must first be able to discriminate between members of a species, subsequently remember the individual’s features and then match the cues stored in its memory with the observed cues of the individual at a later time (Tibbetts and Dale, 2007). Many animals appear to be able to distinguish between members of their own species, which should be beneficial in social interactions such as pair-bonding (Jouventin et al., 2007), attending to offspring (Beecher et al., 1981) and defending territories from unfamiliar intruders (Molles and Vehrencamp, 2001). If animals are able to discriminate between conspecifics, the same cognitive processes may also enable them to discriminate between heterospecifics, such as humans.

Several studies have tested whether wild animals can recognize individual humans. One of the first was conducted on northern mockingbirds (*Mimus polyglottos*): in the experiment, a human repeatedly approached and touched a mockingbird’s nest, thus presenting a salient threat (Levey et al., 2009). Mockingbirds responded to successive approaches by flushing earlier, increasing alarm calling and attacking the intruder. In contrast, their responses to a novel intruder did not differ from those they made in response to the original intruder on their first encounter.

Which features do wild animals use to differentiate between individual humans? Subsequent studies on other bird species have used masks to standardize the appearances of faces and test for discrimination of facial features (Marzluff et al., 2010; Davidson et al., 2015). This may be particularly important in recognizing individual humans, as humans may change their clothing and hairstyles on a frequent basis. Indeed, humans heavily rely on facial features to recognize each other (Maurer et al., 2007). Experiments that used masks to test individual human recognition in free-living American crows have indicated that facial features are important cues in identifying dangerous humans (Marzluff et al., 2010). Interestingly, although crows scolded masks that had been worn during their capture more than they did previously unseen masks, crows also mobbed a person wearing a hat previously paired with a “dangerous” mask in the absence of that mask, suggesting that crows may sometimes use more conspicuous, but changeable, cues rather than identify individual faces.

In another study, American crows were brought into captivity to assess the neural circuitry underlying their responses to familiar human faces (Marzluff et al., 2012). The crows were exposed to one of three stimuli: a human wearing a “threatening” mask that had been worn during the test subjects’ capture, a human wearing a “caring” mask that had been worn while feeding the crows while they were in captivity, and an empty room as a control. Positron emission tomography revealed that the sight of both of the masks activated the rostral forebrain, an area associated with memory and learning (Marzluff et al., 2012). Parts of the amygdala and thalamus, areas associated with fear, were activated more strongly at the sight of the threatening mask than the caring mask. A follow-up experiment that used a human wearing a novel mask as a stimulus, either empty-handed or holding a dead crow, found that certain brain areas, such as the hippocampus and optic tectum, were more strongly activated at the sight of the person with the dead crow, which may facilitate learning of danger (Cross et al., 2013). However, additional control conditions are necessary to determine to what extent the sight of a dead crow itself triggers specific neural activity independent of human presence.

Most of the studies testing individual recognition of humans by wild animals have focused on birds, particularly members of the Corvidae (e.g., Marzluff et al., 2010; Lee et al., 2011; Davidson et al., 2015), a family often described as “feathered apes” because of their comparatively large brains (Emery, 2004; Lambert et al., 2019). However, a study of feral pigeons (*Columbia livia*) in an urban park indicated that this species may also have the ability to recognize individual humans (Belguermi et al., 2011). The experimenters counted the number of pigeons feeding next to a “hostile” and “friendly” human, where the hostile human had interrupted and chased away pigeons in the training sessions, while the friendly human had kept still and allowed the pigeons to feed. Pigeons discriminated between the “hostile” and “friendly” human, even when the experimenters switched locations and coats, suggesting that pigeons may have been using facial cues. If so, this would show that corvids are not unique among birds in recognizing human facial features. This may not be surprising considering the results of a study on honeybees (*Apis mellifera*), which found that these insects were able to discriminate between images of different humans’ faces, and later recognized the target face with a high degree of accuracy (Dyer et al., 2005). This indicates that a capacity to learn human facial features is not limited to the comparatively large and complex brains of vertebrates.

It may be expected that only species or populations that have historically been in regular contact with humans would have an ability to recognize individual humans. A study of Antarctic skuas (*Stercorarius antarcticus*) suggests that this may not be the case (Lee et al., 2016). Skuas on King George Island, which has been colonized by humans only relatively recently, were repeatedly approached at their nests by one of two “intruders.” On the fourth visit, the intruder was joined by a neutral human, whom the skuas had not seen before, and both wore identical clothes. The experimenters walked in opposite directions away from the nest and recorded which person the skuas followed. All seven skua pairs tested chased after and attacked the intruder rather than the neutral human. This study shows that an evolutionary history of living alongside humans does not appear to be necessary for discrimination of individuals, and suggests that the ability to recognize individual humans could be a general ability originating from a need to recognize individual conspecifics. However, it remains to be shown whether wild animals that are completely naïve to humans would be able to discriminate between individuals.

A study of house sparrows provides evidence that the ability to recognize individual people may not arise from extensive experience with humans (Vincze et al., 2015). Subjects were brought into captivity from the wild, from locations designated “urban” and “rural” according to human population density. They were then exposed to an experimenter wearing different masks. The “hostile” mask was paired with a simulated attack from behind the bars of their cages, while the “non-hostile” mask was worn for encounters where the experimenter stayed still in front of the cage. An unfamiliar mask was also used in the test trials, where the sparrows’ risk-taking behavior in response to each mask was quantified. Contrary to the authors’ expectations, sparrows from rural but not urban locations showed a difference in response to the hostile and non-hostile masks, with rural sparrows taking more risks in the presence of the non-hostile mask. While this finding might suggest that urban sparrows do not have the ability to recognize individual humans, it may more likely be a result of other factors such as a difference in boldness, particularly as rural sparrows were more risk-averse than urban sparrows when exposed to the unfamiliar mask. It is therefore important to consider variation among subjects when studying their responses to human cues (see “Variation in responses to humans”).

### “True” Individual Recognition?

What may appear to be individual recognition, i.e., discrimination and memory of an individual’s unique cues, could result from discrimination at the class level (as described in the previous section). For example, a parent may recognize their offspring as their own, but not be able to distinguish among members of their litter or clutch. Similarly, a wild animal may distinguish between a choice of two humans, but not from a wider selection of humans. If subjects respond to only one of the individuals featured in the experiment, it is unknown whether the subjects are responding to the individual rather than a particular cue or set of cues that may be shared by other individuals that exist outside the experimental setup (see e.g., Tibbetts and Dale, 2007; Proops et al., 2009).

To find out whether animals are responding to specific individuals, rather than exhibiting a generalized response to a group of individuals with shared or similar features, some researchers have recommended testing whether animals integrate cues from different sensory modalities, such as visual and auditory cues (Proops et al., 2009; Yorzinski, 2017). In studies of cross-modal recognition, a cue associated with one individual in one sensory mode (e.g., the sight of a familiar individual’s face) is paired with a cue of another individual in a different sensory mode (e.g., a different individual’s voice) to create an “incongruent” stimulus. Animals that are able to integrate both types of cue to form a mental representation of an individual are expected to show signs of expectancy violation when cues from two different individuals are presented together. Therefore, animals may look longer at the incongruent stimulus compared to a congruent stimulus consisting of two cues from the same individual. Such behavior indicates that the subject has an internal representation of the individual and thus recognition must be at the individual rather than class level. The cross-modal experimental paradigm has been used to show individual recognition of conspecifics by free-living African lions (visual-auditory, Gilfillan et al., 2016), but whether wild animals could cross-modally recognize individual humans remains unknown. As it requires animals to be familiar enough with individual humans to recognize them with more than one sense, it may not be likely.

The converse of the problem of whether animals are truly recognizing individuals, rather than classes, is whether a lack of appropriate behavioral response is truly indicative of an inability to discriminate between individuals. An animal may be able to perceive and remember differences between individual humans, but generalize an encounter with one human to all or a wider set of humans. As yet, the conditions under which wild animals generalize from encounters with humans are unknown. The number of previous encounters with humans, the number of different humans encountered and their perceptual similarity could potentially affect how animals respond to an unfamiliar human. This may be particularly important in understanding the effects of feeding interactions. If animals generalize from their experiences of being given food by rewarding humans, they may be more inclined to approach unfamiliar humans and be at risk of being harmed by dangerous humans. Research in this area would therefore be valuable.

### Social Learning About Dangerous Individual Humans

In many species, information about danger can spread through a population by social learning, often through observational conditioning (Griffin, 2004). This can be facilitated by exposure to conspecific alarm calling and mobbing the threatening stimulus, usually a predator. Alarm calls function to alert other individuals in the vicinity to danger, and alarm calling can cause an otherwise innocuous stimulus to be perceived as a threat (Curio et al., 1978). Following up on the finding by Marzluff et al. (2010) that American crows remember people that have previously captured them, Cornell et al. (2011) tested whether this information subsequently spreads to conspecifics. They found that, even 5 years after the capture event, crows continued to scold the dangerous mask to a greater extent than the neutral mask. The increasing number of crows scolding over time, combined with scolding by lone crows that had never been captured, indicated that the stimulus had been learned socially *via* observational conditioning, with the sight and sound of conspecifics scolding allowing naïve crows to learn the association. A study of another corvid, the Eurasian jackdaw (*Corvus monedula*), found that just the sound of conspecifics scolding was sufficient to cause a change in behavior toward a human wearing a particular mask (Lee et al., 2019): jackdaws returned to their nest-boxes more quickly when confronted with the “scolding” mask compared to a previously-seen neutral mask. These experiments highlight the potential benefit of learning the cues of individual humans through social means: a subject need not experience a dangerous encounter with a human in order to learn to avoid the same human in later encounters, which could have considerable implications for survival.

## Variation in Responses to Humans

In the previous sections, we outlined how cognitive processes influence the responses of wild animals to encounters with humans. However, not all animals respond to humans in the same way, and considerable variation exists both between and within species. Understanding the causes and consequences of this variation is important, as it may influence the ability of animals to persist in habitats dominated by anthropogenic activity (Sih et al., 2011; Lowry et al., 2013; Sol et al., 2013). In this section, we discuss how wild animals vary in their responses to humans, the proximate mechanisms underlying this variation, and its wider ecological implications. We then outline how an understanding of the cognitive processes underlying responses to humans, and the interactions of these processes with other traits, can be applied to help address urgent conservation and wildlife management problems.

### Why do Animals Vary in Their Responses to Humans?

Variation in responses to humans may arise if animals differ in their perception of cues, their previous experience and/or their behavioral decision-making processes (Sih et al., 2011; see previous sections). Variation can arise at each of these stages: for example, while animals may perceive relevant cues and classify them in a similar way, differences in prior experience may result in behavioral variation (Sih et al., 2011; Greggor et al., 2014, 2019). Firstly, an animal’s response to a cue is likely to depend on the specificity of the cue itself, and how reliably it predicts a particular outcome (Shettleworth, 2010). The animal’s subsequent behavioral response may then be based on the context-specific payoff of potential outcomes; for instance, animals may decide to ignore a cue signaling a mild threat if fleeing incurs a substantial energy cost (Sih et al., 2011). Responses to novel cues may further depend on how closely cues match those encountered in an animal’s evolutionary past or previous experience, which may have generated a cognitive or perceptual bias for certain types of information. For example, wild animals may be more likely to attend to human gaze cues if they frequently attend to the gaze direction of conspecifics (see Davidson et al., 2014 for a discussion), or they may employ social learning to avoid dangerous people if they rely heavily on social learning in other contexts. Additionally, individual-level factors such as personality, response to novelty, reproductive state and previous experience also influence how individuals use information from their environment (Sih and Del Giudice, 2012; Greggor et al., 2017, 2019; Figure 1), and are therefore likely to contribute to decision-making during encounters with people. Although there is growing interest in how cognitive variation influences responses to human-induced rapid environmental change in general (e.g., Greggor et al., 2014, 2019; Barrett et al., 2019), relatively few studies have focused specifically on the role of cognition in determining how animals respond to humans themselves.

To date, the majority of studies investigating behavior during human-wildlife encounters has focused on quantifying differences between animal populations in habitats that differ in the level of human disturbance, such as along urban-rural gradients (e.g., Samia et al., 2015; Gaynor et al., 2018; Breck et al., 2019). While animals living in urban habitats are typically less fearful of humans than their rural counterparts, the mechanisms driving this variation remain relatively poorly understood (Sol et al., 2013). It is possible that urban environments select for individuals with particular traits that enhance survival and reproductive success (natural selection), or that individuals with certain traits are more likely to colonize urban habitats in the first place (non-random sorting). Perhaps the more common (though not mutually exclusive) scenario is that animals living in urban environments adjust their behavior over time *via* learning, or other forms of behavioral plasticity (Sol et al., 2013). These behavioral adjustments may take many forms, including altering habitat use to minimize contact with people (Duarte et al., 2011; Bonnot et al., 2020), or becoming more tolerant of human presence through habituation and/or risk allocation (Lima and Bednekoff, 1999; Rodriguez-Prieto et al., 2009). Whether animals tolerate or avoid human disturbance is likely to depend on the nature of their interactions with people. For example, eastern gray kangaroos (*Macropus giganteus*) flee more readily from humans in areas with higher hunting pressure, compared to those in areas with a higher density of tourists and other forms of non-lethal disturbance (Austin and Ramp, 2019). Conversely, animals may approach humans in areas where this behavior is actively rewarded: Barbary macaques (*Macaca sylvanus*) appear to spend more time using roadside habitat where they are provisioned by tourists, especially at times of higher tourist activity and when natural food sources become scarce (Waterman et al., 2019). Currently, most studies in this area focus on how animals make escape decisions during encounters with humans. As a result, less is known about how animals come to associate people with reward as opposed to danger. In areas where wild animals encounter humans that vary in their level of threat, animals may benefit from using human cues to assess risk, categorizing people based on risk level, and discriminating between individual humans (see previous sections).

Even within the same habitat, individuals may differ in their behavior during encounters with humans. For example, burrowing owls (*Athene cunicularia*) show individual consistency in flight initiation distance (Carrete and Tella, 2013), roe deer react in a moderately repeatable way to capture and handling (Bonnot et al., 2015), and yellow-bellied marmots (*Marmota flaviventris*) differ in their rates of habituation to humans (Runyan and Blumstein, 2004). While an individual’s previous experience is likely to inform their decision-making, personality differences may also contribute to the observed variation in responses. Personality, which refers to consistent inter-individual differences in behavior, is widespread in the animal kingdom (Bell et al., 2009; Sih et al., 2012). These behavioral differences influence animals’ responses to novel resources or habitats (e.g., Kozlovsky et al., 2017; Lapiedra et al., 2017; Thompson et al., 2018; Breck et al., 2019) and novel threats (Short and Petren, 2008; Lapiedra et al., 2018). Suites of behaviors may be correlated across contexts in a behavioral syndrome (Sih et al., 2004), potentially influencing how individuals respond to ecological change (Dingemanse et al., 2004; Sih et al., 2012; Lapiedra et al., 2017).

Individuals may also differ in how they gather and process information during decision-making; while explaining inter-individual differences in cognitive ability is a topic of growing research interest (Boogert et al., 2018; Cauchoix et al., 2018), it is not known how cognitive variation influences behavior during human-wildlife encounters. Furthermore, it is highly likely that personality interacts with cognition to determine how individuals respond to humans. For example, individuals’ exploratory tendencies may influence their exposure to cues in the environment, and also opportunities for learning (Sih and Del Giudice, 2012). While intriguing, the relationship between personality and cognitive ability is currently poorly understood and is likely to be complex, potentially varying between populations and habitats in a context-dependent manner (Dougherty and Guillette, 2018). Regardless of the exact mechanisms involved, the fact that individuals appear to differ in their responses to human encounters raises the possibility that some individuals may be better able to cope with the challenge of living alongside humans. If these behavioral differences are heritable and enhance fitness, this could result in long-term evolutionary change (Sol et al., 2013).

Though empirical studies are currently limited, there is some evidence to suggest that animals’ responses to human disturbance may influence survival and reproductive success. For example, elk and brown bear (*Ursus arctos*) show consistent individual differences in their tolerance of human disturbance, which influences habitat use during the hunting season; consequently, individuals that spend more time near roads are more frequently seen and killed by hunters (Ciuti et al., 2012a; Leclerc et al., 2019). A study of spotted hyenas (*Crocuta crocuta*) also suggests that individuals that take more risks when foraging are less likely to survive to adulthood (Greenberg and Holekamp, 2017); in this case, responses to humans were not investigated explicitly, but the findings indicate that differences in risk-taking tendencies may have important implications for survival in anthropogenic habitats. These examples illustrate how humans, through our lethal and non-lethal interactions with wildlife, may exert selective pressure on cognition and behavior. While the mechanisms underpinning animals’ responses to humans are not well understood, their impacts have potentially far-reaching consequences for evolutionary processes and population dynamics, as discussed in the next section.

### Wider Implications

Although human activity has been shown to exert strong selective pressure on wildlife (Hendry et al., 2008; Darimont et al., 2009), how direct encounters with humans shape animal cognition and behavior is poorly understood. Identifying the factors that influence animal decision-making, and their fitness consequences, may shed light on why some species (or individuals) are more successful than others in exploiting human-dominated habitats. In particular, we can begin to determine: (i) the extent to which individuals change their responses to humans within their lifetime, and the cognitive processes involved (plasticity), (ii) whether individual variation in human-disturbed habitats reflects the behavioral variation at the species level, or whether these individuals represent a “subset” of the population (non-random sorting), and (iii) the extent to which these behaviors are heritable, and contribute to individual fitness (natural selection; Sol et al., 2013). Furthermore, we can begin to investigate how these processes interact with factors such as life history to influence population persistence (Sol et al., 2013; Maspons et al., 2019). How behavior and life history interact to influence survival in changing environments is not well understood, but current evidence suggests that the value of behavioral plasticity may be higher for species with long lifespans and comparatively low rates of reproduction (Maspons et al., 2019). Thus, processes such as learning could buffer populations against the effects of maladaptation and enhance survival under rapidly-changing conditions (Maspons et al., 2019).

In addition to influencing species persistence, how wild animals respond to encounters with humans may affect population dynamics and community composition (Schlesinger et al., 2008; Tuomainen and Candolin, 2011; Pirotta et al., 2018). For example, the extent to which animals tolerate or avoid humans is likely to influence habitat use (Rodríguez-Prieto and Fernández-Juricic, 2005; Mallord et al., 2007), leading to local changes in species abundance and richness (Mallord et al., 2007; Bötsch et al., 2017, 2018). As a result, these changes may modify interactions between predators and prey (e.g., Berger, 2007; Gaynor et al., 2018; Bonnot et al., 2020). Changes in predator-prey interactions may have wider population-level impacts: for instance, puma (*Puma concolor*) respond to human disturbance by reducing feeding time at individual kills, but appear to compensate for this reduced energy intake by killing more deer in areas of higher human population density (Smith et al., 2015). How wild animals respond to encounters with humans may therefore not only influence individual fitness, but the composition and persistence of entire communities, with implications for conservation and the mitigation of human-wildlife conflict.

## Conservation and Management Applications

A clearer understanding of how animals respond to encounters with humans could be applied to mitigate the impacts of anthropogenic activity. Problems can arise when animals exhibit inappropriate responses to humans. For example, failing to habituate to non-threatening human disturbance may compromise fitness, through increasing stress levels (Ellenberg et al., 2007, 2009) or leading animals to avoid disturbed habitats that are otherwise of suitable quality (an “undervalued resource”; Gilroy and Sutherland, 2007). In some cases, exhibiting the “correct” response during human-wildlife encounters can also be problematic. For instance, habituated animals may exploit anthropogenic food sources that are easy to obtain, but that compromise health (Waterman et al., 2019) or bring them into conflict with humans (Breck et al., 2019; Goumas et al., 2019). In the latter case, some individuals may present a greater cause for concern than others due to their reduced fear of humans (“problem” individuals; Swan et al., 2017). Likewise, the deleterious effects of human disturbance may disproportionately impact certain individuals, depending on factors such as temperament or reproductive state (Dyck and Baydack, 2004; Ellenberg et al., 2009). Knowledge of the proximate and ultimate mechanisms underlying variation in responses to human-wildlife encounters is therefore valuable in deciding whether specific individuals or groups need to be targeted for conservation or management interventions (Swan et al., 2017).

Cognitive research can provide an important tool in mitigating the impacts of human-wildlife interactions (Greggor et al., 2014, 2019; Barrett et al., 2019). Understanding how animals perceive and respond to humans can be used to limit impacts on wildlife populations by creating spatial or temporal “buffer zones” (Rodríguez-Prieto and Fernández-Juricic, 2005; Mallord et al., 2007; Gaynor et al., 2018); encouraging establishment in high-quality habitat (Gilroy and Sutherland, 2007; Greggor et al., 2019); or identifying the factors causing some species or individuals to exploit anthropogenic food sources (Swan et al., 2017; Barrett et al., 2019). A conceptual framework developed by Greggor et al. (2014) outlines how the problems caused by human-induced rapid environmental change can be mitigated by identifying the relevant perceptual and cognitive mechanisms underlying behavior. Applied in the context of direct encounters between humans and wildlife, key questions arise at the following levels: (i) perception – which human cues facilitate animal decision-making, and how are these cues perceived and categorized by the animal in question?; (ii) learning – how does experience influence decision-making, and what are the cognitive processes involved? Once the relevant cognitive and perceptual processes have been identified, they can be targeted to achieve (iii) the desired change in behavior (Greggor et al., 2014, 2019). Fundamental cognitive research has already been instrumental in helping to solve some conservation problems (e.g., O’Donnell et al., 2010; Urbanek et al., 2010); but few studies have applied this framework to manipulate behavioral responses to humans themselves, though some progress is being made. For example, recent experiments with urban herring gulls (Goumas et al., 2019, 2020) show that these birds use gaze and other human behavioral cues when selecting anthropogenic food, suggesting that simple changes in human behavior could help to reduce conflict between humans and herring gulls in urban areas.

## Future Directions

In light of unprecedented rates of environmental change, further research into the responses of wild animals to encounters with people is urgently needed. Controlled experiments can be effectively used to elucidate the cognitive mechanisms underpinning wild animals’ responses to humans, both across a range of habitats that vary in their frequency of human-wildlife encounters and where humans present varying levels of threat. Long-term field studies, where individuals can be accurately identified and monitored over time, are particularly valuable in this regard. Firstly, by experimentally manipulating the various aspects of human-wildlife encounters, we can identify relevant cues involved in risk assessment; how these cues are perceived and categorized; how previous experience shapes decision-making, and how information about people is transmitted through populations (Cornell et al., 2011; Sih et al., 2011). Secondly, by monitoring the behavioral responses of known individuals over time and across contexts, we can begin to determine how and why individuals differ in their responses to people. Response measures could be complemented with assays of personality and cognitive ability (Sih and Del Giudice, 2012; Dougherty and Guillette, 2018), in order to examine how these factors interact to influence decision-making. While the question of how cognition interacts with personality to determine behavior is attracting growing interest, relationships uncovered to date suggest a complex picture (Dougherty and Guillette, 2018). Field studies that generate robust measures of personality and cognitive ability from individually-identifiable animals, in a range of habitats and contexts, would therefore be extremely valuable.

Studies of animals living in human-dominated habitats must be complemented by studies of their rural counterparts, in order to determine the selection pressures acting on behavior. Although recent years have seen a proliferation of studies quantifying behavioral differences along urban-rural gradients, few studies identify the relevant mechanisms involved in generating this variation. Moreover, with the exception of studies examining the role of neophobia and categorization (Greggor et al., 2016), the cognitive processes underlying urban-rural differences in behavior have been largely overlooked. While the majority of evidence to date supports behavioral plasticity as the main driver of urban-rural differences in behavior, non-random sorting may be relevant in cases where dispersal propensity and tendency to colonize urban habitats covary with other behavioral traits. Natural selection may also be at work if behavior is heritable and contributes to individual fitness (Sol et al., 2013). Finally, by identifying the fitness consequences of individual variation in responses to human disturbance, we can also begin to investigate the broader ecological consequences. To this end, studying organisms with different life-history strategies (Maspons et al., 2019) and occupying a range of trophic levels would allow researchers to uncover how wild animals’ responses to humans affect population dynamics, species distributions and community composition.

While the recommendations outlined above are ambitious, expanding research on the responses of wildlife to human encounters can play a pivotal role in reducing the impacts of human activity. Many long-term, individual-level behavioral studies already exist around the world, which are likely to provide suitable systems for investigating these types of questions. Studies covering a range of different species and habitats, where humans vary in the nature of their relationship with wildlife, provide an opportunity for “natural experiments” to identify how interactions with humans shape animal cognition and behavior. It would be particularly interesting to carry out these studies in areas experiencing relatively recent and/or rapid expansions of human activity. However, given that all habitats on Earth are now impacted by human activity to some degree (Ellis, 2011; Waters et al., 2016), it can often be difficult to obtain accurate information about wild animals’ previous exposure to humans. To this end, simulation models supported by empirical evidence may be particularly useful in improving our understanding of the anthropogenic pressures facing wildlife around the world and their long-term consequences. Finally, we would encourage the publication of all studies, including null results and contradictory findings, in order to refine methodologies, quantify empirical support for existing theory and develop new theoretical frameworks, and improve the reliability of results (van Assen et al., 2014).

## Conclusion

Living alongside humans is a challenge for many wild animals, particularly in scenarios where people differ in their behavior toward wildlife. How wild animals respond during encounters with humans is likely to be controlled by a range of cognitive processes, and may carry important fitness consequences. In this review, we have considered the role of animal cognition in human-wildlife encounters, and its important influence on the ability of individuals, populations and species to cope with life in a human-dominated world. Further research in this area is vital to identifying the selection pressures on animal cognition associated with human-induced ecological change, and would assist in mitigating the negative impacts of human activity.

## Author Contributions

MG and VL co-wrote the manuscript with guidance and significant input from NB, LK, and AT. All authors contributed to the article and approved the submitted version.

### Conflict of Interest

The authors declare that the research was conducted in the absence of any commercial or financial relationships that could be construed as a potential conflict of interest.

## Figures and Tables

**Table 1:** 

Appetitive behavior/stimulus	Characterized by attraction as a result of an animal’s requirement to meet bodily needs
Associative learning	Learning that results from experiencing contingencies, or predictive relationships, between events (Shettleworth, 2010)
Aversive behavior/stimulus	Characterized by repulsion as a result of an animal’s requirement to avoid harm
Behavioral plasticity	The ability of animals to formulate behavioral responses to cope with new or unusual challenges (Ducatez et al., 2020)
Categorization	An organism’s ability to respond equivalently to members of the same class, to respond differently to members of different classes, and transfer those responses to novel, discriminably different members of these classes (Lazareva and Wasserman, 2010)
Class-level recognition	(i) Receivers learn the signaller’s individually distinctive characteristics and associate these characteristics with inferred class-specific information about the signaller; and (ii) receivers match the signaller’s phenotype to an internal template associated with different classes (Tibbetts and Dale, 2007)
Discrimination	The act or process of distinguishing between stimuli or of recognizing or understanding the differences between things (Colman, 2008)
Generalization	The tendency for a learned response to a particular stimulus to be elicited by other stimuli that resemble it (Colman, 2008)
Habituation	A form of non-associative learning involving decreased responsiveness to a stimulus with repeated presentation (Blumstein, 2016)
Individual recognition	A subset of recognition that occurs when one organism identifies another according to its individually distinctive characteristics (Tibbetts and Dale, 2007)
Observational conditioning	Associating a cue or object with an affective state or behavior(s) by virtue of watching demonstrators respond to it (Shettleworth, 2010)
sensitization	A form of non-associative learning involving increased responsiveness to a stimulus with repeated presentation (Blumstein, 2016)
Social learning	Learning that is influenced by observation of, or interaction with, another individual or its products (Heyes, 1994)
